# Synergistic effects of the sesquiterpene lactone, EPD, with cisplatin and paclitaxel in ovarian cancer cells

**DOI:** 10.1186/s13046-015-0157-2

**Published:** 2015-04-25

**Authors:** Caroline van Haaften, Arnoud Boot, Willem E Corver, Jaap DH van Eendenburg, Baptist JMZ Trimbos, Tom van Wezel

**Affiliations:** Department of Gynecology, Leiden University Medical Center, Albinusdreef 2, 2333 ZA Leiden, The Netherlands; Department of Pathology, Leiden University Medical Center, Albinusdreef 2, 2333 ZA Leiden, The Netherlands

**Keywords:** Ovarian cancer, EPD, Cisplatin, Paclitaxel, Synergism

## Abstract

**Background:**

Ovarian cancer remains still the leading cause of death of gynecological malignancy, in spite of first-line chemotherapy with cisplatin and paclitaxel. Although initial response is favorably, relapses are common and prognosis for women with advanced disease stays poor. Therefore efficacious approaches are needed.

**Methods:**

Previously, an anti-cancer agent, EPD exhibited potent cytotoxic effects towards ovarian cancer and not towards normal cells. Cell viability and cell cycle analysis studies were performed with EPD, in combination with cisplatin and/or paclitaxel, using the ovarian carcinoma cell lines: SK-OV-3, OVCAR-3, JC, JC-pl and normal fibroblasts. Cell viability was measured using Presto Blue and cell cycle analysis using a flow cytometer. Apoptosis was measured in JC and JC-pl , using the caspase 3 assay kit.

**Results:**

In JC-pl, SK-OV-3 and JC, synergistic interactions between either EPD and cisplatin or EPD and paclitaxel were observed. For the first time the effects of EPD on the cell cycle of ovarian cancer cells and normal cells was studied. EPD and combinations of EPD with cisplatin and/ or paclitaxel showed cell cycle arrest in the G_2_/M phase. The combination of EPD and cisplatin showed a significant synergistic effect in cell line JC-pl, while EPD with paclitaxel showed synergistic interaction in JC. Additionally, synergistic drug combinations showed increased apoptosis.

**Conclusions:**

Our results showed a synergistic effect of EPD and cisplatin in an ovarian drug resistant cell line as well as a synergistic effect of EPD and paclitaxel in two other ovarian cell lines. These results might enhance clinical efficacy, compared to the existing regimen of paclitaxel and cisplatin.

**Electronic supplementary material:**

The online version of this article (doi:10.1186/s13046-015-0157-2) contains supplementary material, which is available to authorized users.

## Introduction

Ovarian cancer, the silent killer, is the leading cause of death from gynecological malignancy. In 2012 it was diagnosed at an incidence of 1.3 cases per 10.000 inhabitants in the Western world, while in the USA 22,280 new cases were diagnosed. An estimated 15,500 women will die from the disease per year (the American Cancer Society). This cancer is mostly manifested at advanced stage (Stage III, IV), having spread beyond the ovaries to involve the peritoneal (abdominal) cavity. The serous papillary variants (>70%) of the ovarian epithelium form the largest subgroup [1]. With the current chemotherapy, using platinum derivates and/or paclitaxel before or after surgery, most patients respond favorably. However relapses are common after this first line treatment. Relapses are most likely caused by a subset of tumor cells that have the ability to survive after completion of therapy [2,3]. So far the prognosis for women with advanced disease remains poor and more efficacious approaches are badly needed. The greatest challenge will be to craft combinations of therapy that result in years of improved survival [4]. Paclitaxel is an agent isolated from the bark of the western yew tree *Taxus brevifolia*. It is an anti-microtubule agent stabilizing tubulin polymerization that causes cell arrest in the G_2_ and M phases of the cell cycle*.* Cisplatin is a chemotherapeutic drug that causes cross linking of DNA, which ultimately triggers apoptosis [5-7]. Previous research has identified EPD as a potential new anti-cancer agent [8]. EPD, eremophila-1(10)-11(1230-dien-12,8β-olide of the eremophilanolide structure subtype [9], has been isolated from *Calomeria amaranthoides* of the family Asteraceae (Compositae). This agent, a sesquiterpene lactone (SL), has been found to exhibit potent cytotoxic effects towards ovarian cancer cells *in vitro* and *in vivo* [8,10]. SLs have been reported as being anti-cancer as well as anti-inflammatory agents. The majority of SLs are derived from the family Asteraceae. SLs are colorless and natural bitter compounds of the subfamily of terpenoids, with lipophilic character. This lipophilicity can facilitate penetration through the cell membrane, causing increased SL cytotoxicity *in vitro*. To date several SLs are used in ongoing clinical trials, such as Artemisinin, Dimethyl-amino-parthenolide and Thapsigargin [11,12].

To further study the effects of EPD, *in vitro* growth inhibition studies with EPD, cisplatin, paclitaxel and in combination, were performed with ovarian cancer cell lines and normal skin fibroblasts.

## Materials and methods

### Agents

EPD has been provided by the department of Pharmacy, Sydney University, NSW, Australia. In short: Fresh leaves of *Calomeria amaranthoides,* were steam distillated to get a high recovery of sesquiterpene-rich oil. The oil was fractionated by short-column vacuum chromatography to establish 95% purity of EPD [10]. Paclitaxel and cisplatin were obtained from Sigma-Aldrich, USA.

### Cell culture

Cell lines (of the serous subgroup) used for the assays were JC and JC- pl [13], OVCAR-3 and SK-OV-3 (both from the American Type Culture Collection (ATCC)). Normal human skin fibroblasts were provided by the department of Dermatology, LUMC, The Netherlands. The cell lines were grown in RPMI-1640, supplemented with 2 mM L-Glutamine (Gibco, Invitrogen, UK), 10% heat inactivated fetal calf serum (FCS) (Sigma), penicillin (50 units/mL) and streptomycin (50 μg/mL) (Invitrogen, UK). Normal skin fibroblasts were grown in Dulbecco’s modified Eagle medium (DMEM) (Invitrogen, UK), also supplemented with L-glutamine and 10% FCS. The cultures were maintained in an incubator with humidified atmosphere at 37° C with 9% CO_2_. The four human ovarian cancer cell lines were tested for their identity profile (ID) using a Cell ID™ kit from Promega. SK-OV-3 and OVCAR-3 were compared to their known profile of the ATCC and JC and JC-pl were cross referenced.

### *In vitro* cytotoxicity tests

*In vitro* cytotoxicity tests were performed using a non-fluorescent substrate, Presto Blue (Bio Source, Invitrogen, UK). Cells were seeded at approximately 16,000 cells/cm^2^ in 24-wells plates (Costar, USA) in 1 mL medium/well. After 24 hrs, exponential growing cell cultures were treated in triplicate with the different compounds EPD, paclitaxel and cisplatin in 2 mL/ well fresh medium. Control (Bl) cells were untreated. The cells were incubated with the drugs for 72 hrs to ensure two doubling times of the cells. Cell viability was measured to determine the best doses for the assays. Different concentrations for each agent were used for each ovarian cancer cell line and for the normal fibroblasts. EPD was re-dissolved in dimethyl sulfoxide (DMSO), with final concentration of 0.02% DMSO. Combination of drugs used were: EPD + cisplatin, EPD + paclitaxel; cisplatin + paclitaxel and EPD + cisplatin + paclitaxel. Final concentrations are given in Table 1. After 72 hrs incubation, Presto Blue (10%) was added to the cultures. After 2–3 hrs of incubation the percent of cell viability was measured with a multi plate reader, Victor 3 V (Perkin Elmer) by transferring 100 μl of the medium in a 96 well plate (Greiner Bio-one). Wave lengths used were 570 and 610 nm, with 570/8 and 610/10 filters. Viability was calculated relative to untreated cells. Synergistic drug interactions were evaluated using a simplified version of the Bliss independence model [14].

**Table 1:** **STR profiles of the cell lines: JC and JC-pl**

**Sample**	**THOI**	**D21S11**	**D5S818**	**D13S317**	**D7S820**	**D16S539**	**CSF1PO**	**AMEL**	**vWA**	**TPOX**
JC	6,9.3	28,31	11	8,14	10	11	11	X	17,19	8,11
JC-pl	6,9.3	28,31	11	8,14	10	11	11	X	17,19	8,11

### Statistical analysis

Statistical analyses were performed using SPSS 20.0 (SPSS Inc., Chicago, Ill) to calculate means with standard errors (SE).

### Apoptosis

Cells were seeded as described for the *in vitro* cytotoxicity tests. 24 hrs after seeding the following drug concentrations were added for the cell line JC: 3.15 μg/mL EPD and 0.52 μg/mL paclitaxel, for the cell line JC-pl: 6.5 μg/mL EPD and 3.39 μg/mL cisplatin. Apoptosis was measured after 24 hrs of drug treatment using the Caspase 3 Assay kit (Sigma-Aldrich, Cat.nr CASP3F) according to the manufacturer’s specifications.

### Flow cytometry

The four ovarian cancer cell lines and normal fibroblasts were treated using the same conditions as described for the *in vitro* cytotoxicity assays. Cells were seeded in 25 cm^2^ flasks and after 24 hrs drug treatment was initiated. After 72 hrs incubation with drugs, cells were prepared for flow cytometry. In short: cells were harvested using trypsin/EDTA, counted and transferred to FACS tubes (BD Falcon, Cat no 352052), and centrifuged (500 g) for 5 min in 4°C. Supernatant was removed and 50 μl PBS was added to the cell pellet followed by 450 μl 100% cold methanol added drop-wise under constant swirling. Next, the cell suspensions were put for 20 min in the freezer. Then 500 μl cold PBS/Tw 0.05% was added and the cells were centrifuged (500 g) for 5 min at 4°C. Supernatant was decanted and 1 mL PBA 1.0%/Tw 0.05% (PBA/Tw) was added to the pellets. After centrifuging, supernatant was decanted and 500 μl staining solution (PBA/Tw) containing 0.1% RNase (Sigma -Aldrich) and 100 μM propidium iodide (PI) (Sigma-Aldrich) was added and the pellets vortexed and incubated in a 37°C water bath for 30 min. Cells were kept at 4°C until flow cytometric analysis. In order to standardize the data, untreated cells of the different cell lines were used to calibrate the G_1_ position of untreated and treated cells.

For flow cytometric analysis a LSRII (BD Bio Sciences) was used with a 488 nm laser for excitation. Fluorescence was collected using a 610/20 nm band pass filter. Pulse-processing was used to collect 50,000 single cell events. During data storage, all events were included. Data was analyzed using WinList 7.1 (Verity Software House, Topsham, ME).

## Results

### *In vitro* cytotoxicity tests

To verify cell line identity, short tandem repeat (STR) profiling was performed on the ovarian cancer cell lines. Cell ID of SK-OV-3 and OVCAR-3 matched the profiles of the ATCC, while JC and JC-pl are new and unique cell lines, both from the same patient, JC-pl derived from a pleural effusion after cisplatin treatment. JC and JC-pl showed identical STR profiles, not matching any of the known cell lines from the ATCC, Table 1.

Based on dose response curves (Additional file 1: Figure S1) the following concentrations of EPD, cisplatin and paclitaxel were selected for the four cell lines and the normal fibroblasts, Table 2.

**Table 2:** Drug concentrations used for the ovarian cancer cell lines and normal fibroblasts*

	**SK-OV-3**	**JC**	**JC-pl**	**OVCAR-3**	**Fibroblasts**
EPD	3.0	3.0	5.0	1.25	3.0
cisplatin	2.5	2.0	2.75	0.75	2.5
paclitaxel	0.02	0.375	0.225	0.004	0.375

*Concentrations in μg/mL.

**Figure 1:**
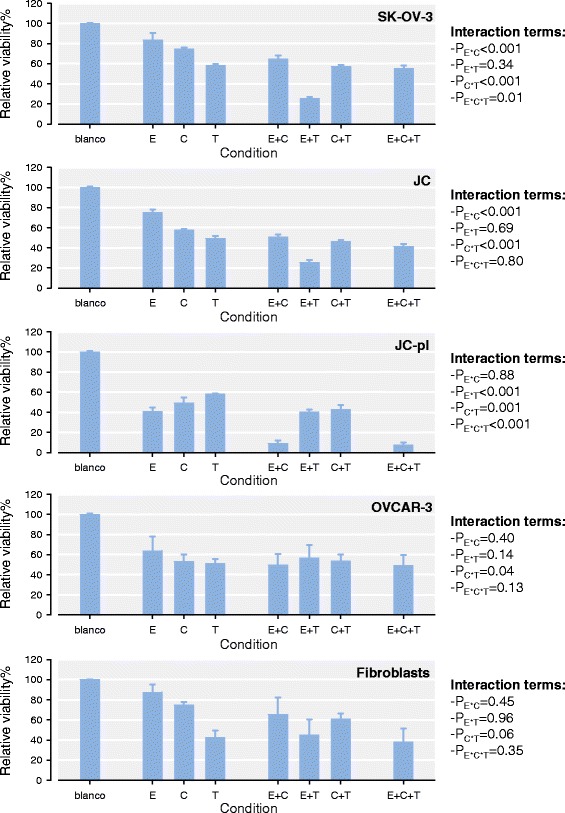
Synergistic effects of combination treatment with EPD on ovarian cancer cell lines and normal fibroblasts. Relative viability is shown for each single compound. E: EPD, C: cisplatin, T: paclitaxel. Synergism was observed between EPD and paclitaxel for SK-OV-3 and JC, whereas for JC-pl the combination of EPD and cisplatin was found to be synergistic.

The relative cell viability of the four cell lines and normal fibroblasts, treated with EPD, cisplatin and paclitaxel are shown in Figure 1. Treatment with EPD, paclitaxel or cisplatin alone resulted in reduced viability in the cell lines, ranging between 41 and 93% of viable cells. Normal skin fibroblasts were affected mostly by paclitaxel after 72 hrs. The combination treatments of EPD with cisplatin and paclitaxel showed increased activity in the different cell lines. To evaluate whether these combination treatments were synergistic drug interactions, a simplified version of the Bliss independence model was applied [14]. When two drugs exert their effects independent to one another, the resulting relative viability after drug treatment is expected to equal the product of the relative viability after treatment with the individual drugs. When the observed viability is higher than the expected based on the individual drug effects, the compounds inhibit each others effects; antagonism. However, when observed viability after combination treatment is lower than the expected viability, then this is an indication of a synergistic drug interaction.

When combining EPD with cisplatin and or paclitaxel, synergistic effects were found. In both SK-OV-3 and JC synergistic effects were found in the combination of EPD and paclitaxel. Treatment of EPD and paclitaxel in SK-OV-3 resulted in cell viability of 93% and 60% respectively. The combined effects were significantly lower than expected for SK-OV-3 with a 25% viability (p < 0.05). The combination of EPD and paclitaxel in JC cells was also found to be synergistic with a resulting viability of 26% (p < 0.05). No synergistic effect was observed when combining EPD and cisplatin in JC. In JC-pl a strong synergistic effect was detected for the combination EPD and cisplatin. Viability of the JC-pl cells for this combination treatment was 9%, where the additive effect of EPD and cisplatin was expected to be 20% (p < 0.05). Paclitaxel with EPD did not have a synergistic effect. OVCAR-3, as well as the normal fibroblasts did not show any synergistic effects with the different combinations. Due to the striking differences in synergistic interactions observed between EPD and paclitaxel in JC and between EPD and cisplatin in JC-pl, the synergistic interactions were further investigated.

### Synergistic interactions between EPD, paclitaxel and cisplatin in JC and JC-pl

**Figure 2:**
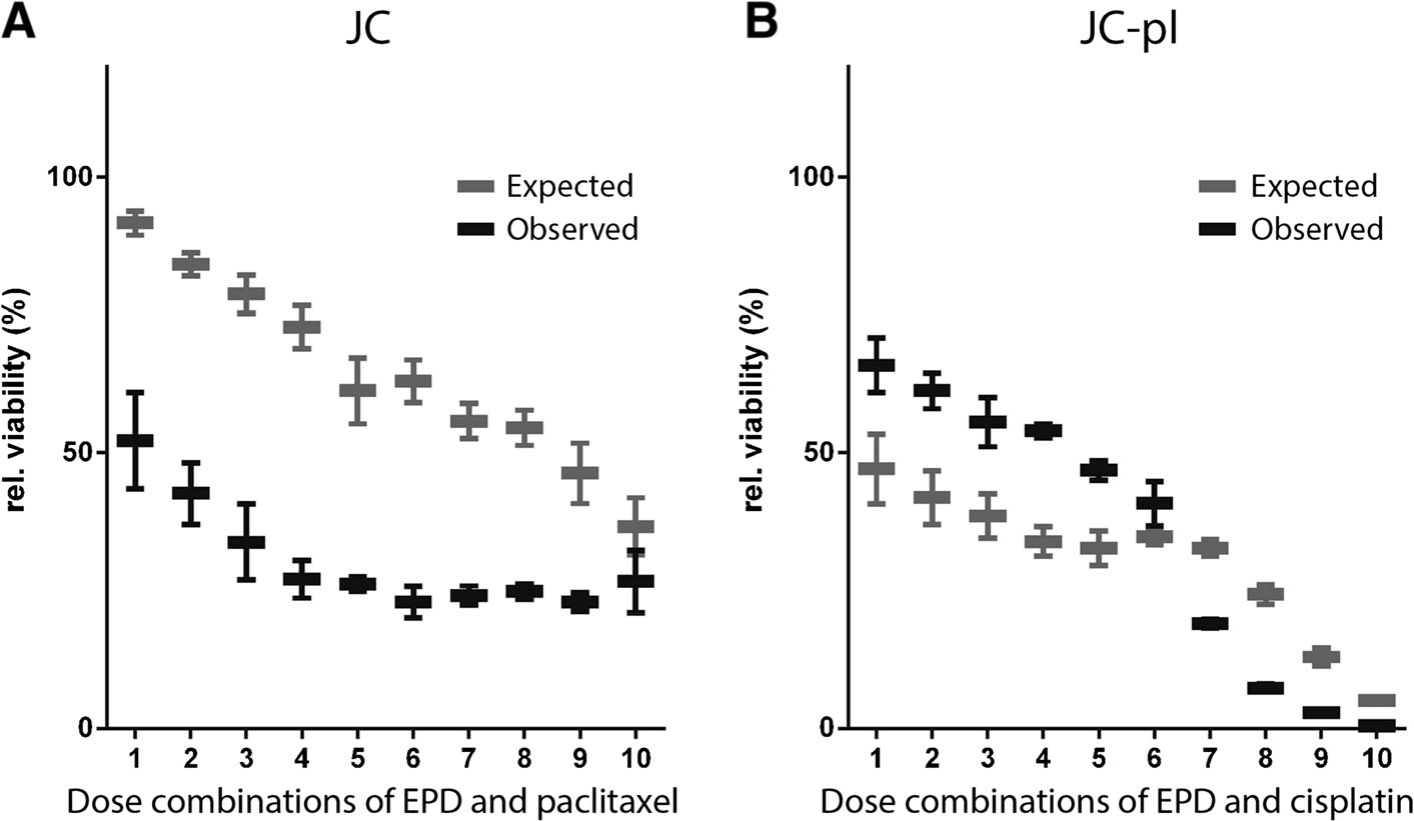
Synergistic drug combinations in JC and JC-pl. **A**: Comparison of expected and observed relative viability of JC after combination treatment with EPD and paclitaxel. **B**: Lower doses of EPD and cisplatin were found to be antagonistic for JC-pl, whereas the higher doses showed synergistic effects.

To further investigate the differences in response to the combinations of drugs in JC and JC-pl, a range of drug combinations was tested for both cell lines. Drug concentrations were selected based on dose–response curves of the individual drugs. Similar to the previous viability tests, using single drug combinations, the expected viability of combination treatment was calculated (Additional file 1: Figure S2). Comparison of the observed viability with the expected viability is shown in Figure 2. For all conditions tested, EPD and paclitaxel showed synergistic effects in JC. For JC-pl the lower concentrations showed an antagonistic drug interaction between EPD with cisplatin. At the highest four doses synergistic interactions were observed, indicating a critical point in the synergistic combinations between these two drugs.

### Synergistic drug combinations induce apoptosis

**Figure 3:**
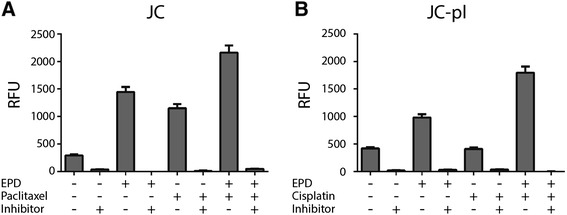
Synergistic drug combinations induce apoptosis. **A**: EPD and paclitaxel in single treatments induced apoptosis. Combination treatment showed an even higher caspase 3 activity. **B**: Cisplatin treatment alone did not induce apoptosis. Combination treatment of EPD and cisplatin showed a strong induction of caspase 3 activity. Inhibitor: Ac-DEVD-CHO; RFU: Relative Fluorescence Units.

To identify the mechanism of growth inhibition of the synergistic interaction between EPD in combination with cisplatin and paclitaxel, apoptosis measurements were performed. Apoptosis was measured using a fluorimetric caspase 3 activity assay. EPD and paclitaxel alone induced apoptosis, cisplatin did not. The combination treatment of JC and JC-pl with EPD and the respective synergistic drug showed an increase in caspase 3 activity (Figure 3). Addition of Ac-DEVD-CHO, a caspase 3 inhibitory small molecule completely abolished the signal in all conditions. This confirmed that the synergistic drug combinations induce caspase 3 mediated apoptosis.

### Flow cytometry

**Figure 4:**
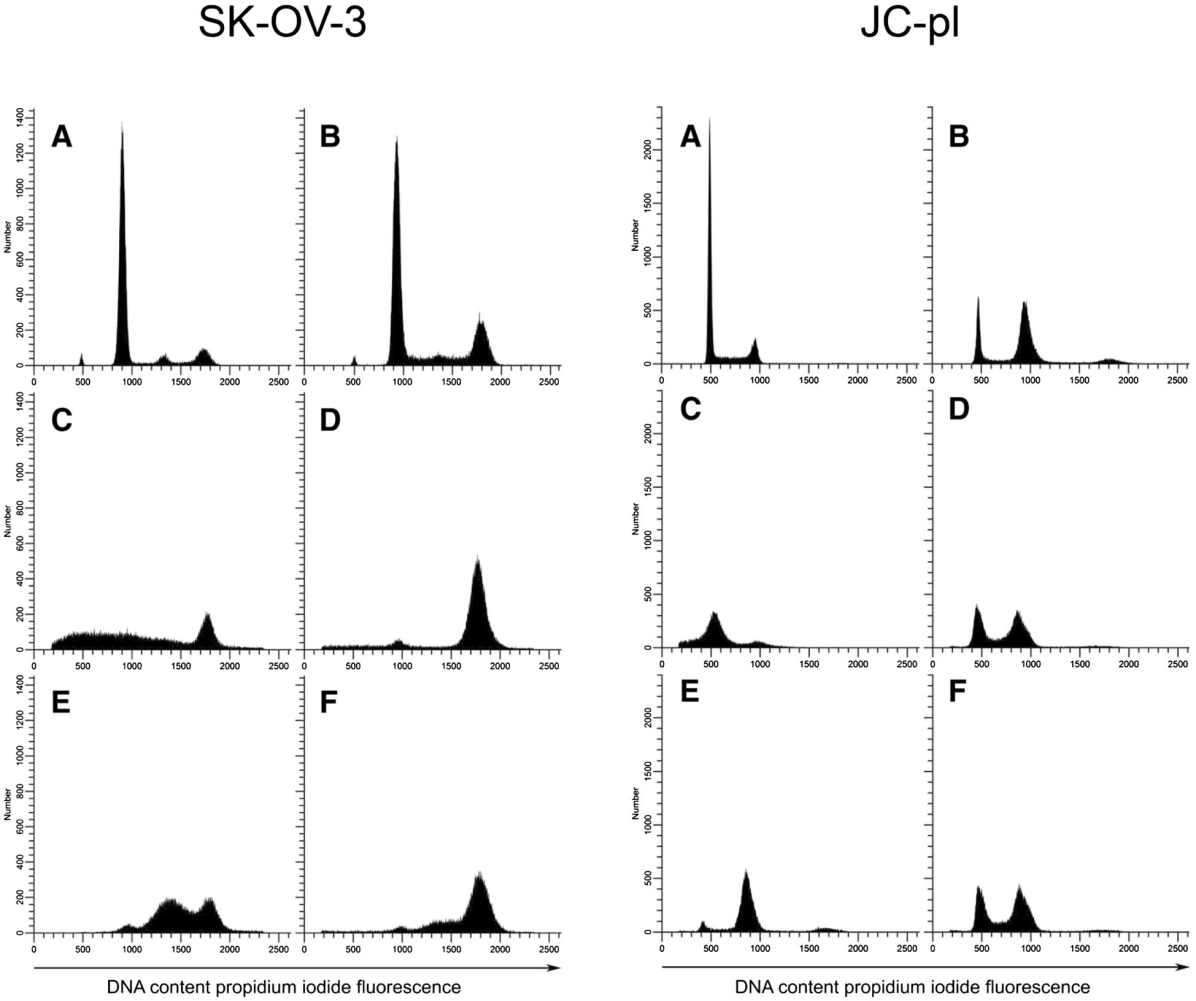
Effects of EPD, paclitaxel and cisplatin on the cell cycle of SK-OV-3 and JC-pl. **4A**: SK-OV-3 treated with: untreated cells **(A)**; EPD **(B)**; paclitaxel **(C)**; EPD + paclitaxel **(D)**; EPD + cisplatin **(E)**; EPD + paclitaxel + cisplatin **(F). 4B**: JC-pl treated with: untreated cells **(A)**; EPD **(B)**; cisplatin **(C)**; EPD + cisplatin **(D)**; EPD+ paclitaxel **(E)**; EPD + cisplatin + paclitaxel **(F)**.

Having established evidence for the synergistic interaction between EPD, cisplatin and paclitaxel in ovarian cancer cell lines, we further sought to elucidate the effects of these compounds. First, the effect of EPD on the cell cycle was studied in SK-OV-3. Untreated SK-OV-3 cells showed to be bi-modal in culture with a minor G_1_ population approximately at (relative) mean channel number (MCN) 501 and a major G_1_ population approximately at MCN 938 (Figure 4A). Already at 3 μg/mL EPD treatment, a clear accumulation of cells in the G_2_/M phase could be noted (Figure 4B). This was dose dependent and G_2_/M cell numbers increased at higher concentrations.

To assess the effects of combinations of compounds on the cell lines therefore we performed cell cycle analysis on all four cell lines as well as normal fibroblasts. Conditions chosen for this experiment were identical to those of the initial cytotoxicity tests. Results are shown in Figure 4. After 72 hrs, 6.25 x 10^5^ cells were counted for SK-OV-3 with EPD treatment and 2.84 x 10^5^ for the combination EPD + paclitaxel, while EPD + cisplatin had 2.89 x 10^5^ cells. Combination of EPD + paclitaxel + cisplatin gave 2.40 x 10^5^ cells. Cell counts of cell line JC-pl with EPD after 72 hrs were: 3.52 x 10^5^, for EPD + cisplatin: 3.40 x 10^5^ and for EPD + cisplatin + paclitaxel: 3.29 x 10^5^ . For all cell lines the percent of viable cells and the number of viable cells per mL was plotted against the viability results from Figure 1 (Additional file 1: Figure S3). Both the percent of viable cells and the number of viable cells per mL showed a strong correlation for JC, JC-pl and SK-OV-3 (Figure 1).

In cell line SK-OV-3 a small peak (MCN = 1439) was noted in the S-phase (Figure 4A), most likely caused by aggregates composed of G_1_ cells of the minor population and G_1_ cells of the major population. Treatment of EPD + paclitaxel resulted in arrest of cells in the G_2_/M phase. Arrest of cells in cell line JC-pl was observed after EPD treatment in the G_1_. The combination EPD + cisplatin showed arrest in G_2_/M, as did the combination EPD + cisplatin + paclitaxel.

**Figure 5:**
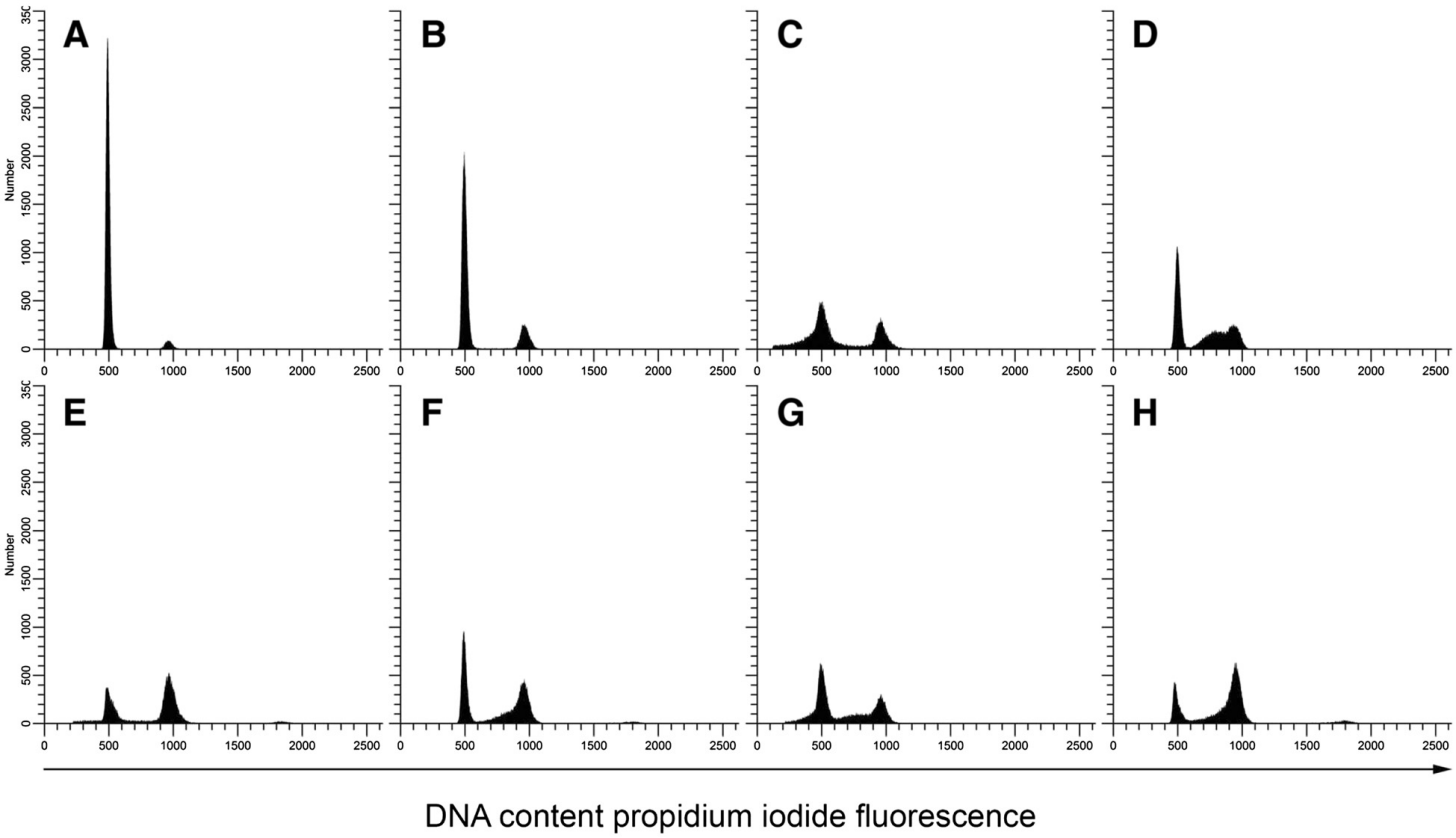
Cell cycle effects of EPD, paclitaxel and cisplatin on normal fibroblasts. **A**: untreated cells; **B**: EPD; **C**: paclitaxel; **D**: cisplatin; **E**: EPD + paclitaxel; **F**: EPD + cisplatin; **G**: paclitaxel + cisplatin; **H**: EPD + paclitaxel + cisplatin.

The effects of EPD on the cell cycle distribution of normal fibroblasts was much less than the effects of paclitaxel and/or cisplatin, but at 3 μg/mL EPD treatment increase in G_2_/M occurred (Figure 5).

## Discussion

Relapses of ovarian cancer in patients with advanced epithelial ovarian cancer is still the main cause of ineffective chemotherapy, most likely due to the development of a drug resistant phenotype [15]. In vitro studies to evaluate the cytotoxicity of taxane and platinum agents have been widely reported the last decades and have demonstrated clinically equal efficacy [16,17]. In our study we have focused on the effects of EPD on four ovarian cancer cell lines, all of the serous subtype.

The sesquiterpene lactone, EPD, has given new evidence to be of great interest as an anti-cancer agent. First the three different compounds EPD, cisplatin, paclitaxel were tested for their cytotoxic effects; both as single drugs and in combination. We demonstrated herein that EPD showed synergistic interactions with paclitaxel (SK-OV-3 and JC) and cisplatin (JC-pl) (Figures 1 and 2). Apoptosis plays an important role in both carcinogenesis as well cancer treatment and can be used as a vehicle of targeted treatment in cancer [18]. Caspase 3 is a frequently activated death protease, causing programmed cell death and has been described in a review article [19]. It is known that many SLs are apoptosis inducers and although SL-induced apoptosis is not fully understood, it is believed that the α-methylene-β-lactone structure is essential for their apoptotic activity [20]. Induction of apoptosis induced by cisplatin and paclitaxel in ovarian cell lines, was earlier measured for OVCAR-3 and SK-OV-3 [21]. Apoptosis induced by EPD was measured for the first time in JC and JC-pl cells, using a caspase 3 assay kit.

Simultaneous treatment of JC with EPD and paclitaxel resulted in increased apoptosis. The combination treatment of JC-pl with EPD and cisplatin was found to be synergistic at the higher doses tested, whilst at the lower doses an antagonistic effect was observed.

JC and JC-pl are new and unique cell lines derived from the same patient. Cell line JC-pl was established after the patient had become resistant to cisplatin and chlorambucil treatment [13]. Interestingly, the cell line which was established after cisplatin treatment showed no decreased sensitivity to cisplatin. Strikingly, JC was found to be sensitive to the combination treatment of EPD in combination with paclitaxel, whereas JC-pl was most effectively inhibited by the combination treatment of EPD with cisplatin. This would suggest that these two cell lines, which are genetically similar, have acquired a somatic alteration resulting in their difference in sensitivity to the tested drug combinations.

EPD might play an important role in combination with cisplatin to resistant cancer cells (Figure 1). To evaluate further the synergistic effects of EPD with paclitaxel and/or cisplatin in the cell lines JC and JC-pl, dose-dependency of synergistic drug interactions was investigated. The cell line OVCAR-3, known for its resistance to chemotherapy [22], did not show synergistic effects with any of the three compounds. Normal skin fibroblasts, showed a small percent decrease in viability at 3 μg/mL, indicative of the low cytotoxicity of EPD. In particular paclitaxel at 0.375 μg/mL had strong cytotoxic effects. Less cytotoxic effects were seen with cisplatin at 2.5 μg/mL (Figure 1), no synergistic effects were found.

In this study four ovarian cancer cell lines and normal fibroblasts treated with EPD were studied for the first time for cell cycle analysis. SK-OV-3 was first tested using a range of concentrations of EPD. It was noted that with increase of EPD > 3 μg/mL the G_0_/G_1_ diminished and the proportion of cells in the G_2_/M-phase increased. Earlier studies concluded that both cisplatin and paclitaxel arrest the cell cycle at G_1_ or G_2_/M. Paclitaxel is known for its development of drug resistance; it is disrupting normal mitotic spindle formation and is arresting cell growth in the M-phase of the cell cycle [23]. Our experiments showed both EPD and paclitaxel resulted in G_2_/M-phase arrest. The effects of EPD combined with cisplatin or paclitaxel showed remarkable changes in the distribution of cells in the G_2_/M-phase (Figure 4). In the flow cytometry analysis of normal skin fibroblasts an increase of G_2_/M in the cell cycle was also noticed after treatment with 3 μg/mL EPD (Figure 5). More studies need to be undertaken to understand the role of EPD, also in combination with cisplatin and paclitaxel.

SLs have demonstrated that their anti-cancer properties are also of interest for their selective targeting of cancer cells, and ability to induce apoptosis [24,25]. Several SLs are currently in cancer clinical trials such as artemisinin, parthenolide and thapsigargin and synthetic derivates have targeted tumor and cancer stem cells, while sparing normal cells [11,12,26,27]. In an *in vivo* experiment the effects of EPD as well as cisplatin on OVCAR-3 cells caused reduction of the abdomen size of mice. However, the mice treated with EPD could be kept for a much longer time than the mice treated with cisplatin [8].

To our knowledge, EPD is the first SL with anti-cancer properties belonging to the subgroup of eremophilanolides. And while in general SLs are thermo labile, EPD can be extracted by steam distillation [10].

There is still a high need for improvement of ovarian cancer treatment. Tomao et al. [28] described in a review article the increasingly important role of ovarian cancer stem cells and the evaluation of alternative models of treatment to overcome the clinical problems of resistance. EPD has shown to be promising in ovarian cancer treatment and as a chemosensitizing agent in combination with other anti-cancer drugs. To date studies are in progress to understand the molecular mechanisms responsible for their anti-cancer activity in ovarian cancers.

## Additional file

Additional file 1:
**Figure S1.** Dose response curves for EPD, cisplatin and paclitaxel for the four cell JC, JC-pl, OVCAR-3 and SK-OV-3. **Figure S2.** Observed relative viability for JC and JC-pl after treatment with single drug and combinations of EPD and paclitaxel (JC) and EPD and cisplatin (JC-pl). **Figure S3.** Percentage of viable cells per mL plotted against the relative viability for SK-OV-3, JC, OVCAR-3 and JC-pl.
