# Supplementary figures and images for: Synergistic effects of the sesquiterpene lactone, EPD, with cisplatin and paclitaxel in ovarian cancer cells
Source: J Exp Clin Cancer Res. 2015 Apr 25;34(1):38. doi: 10.1186/s13046-015-0157-2 (PMC4472250; doi:10.1186/s13046-015-0157-2)

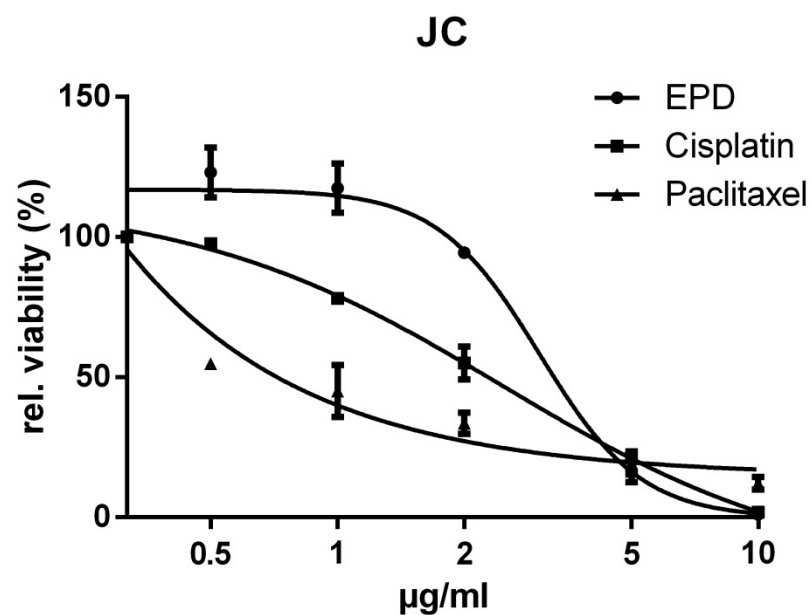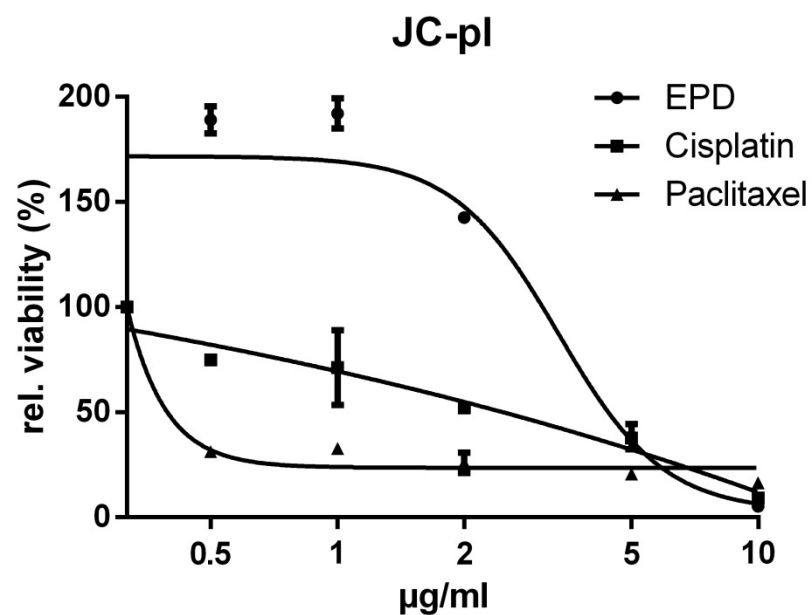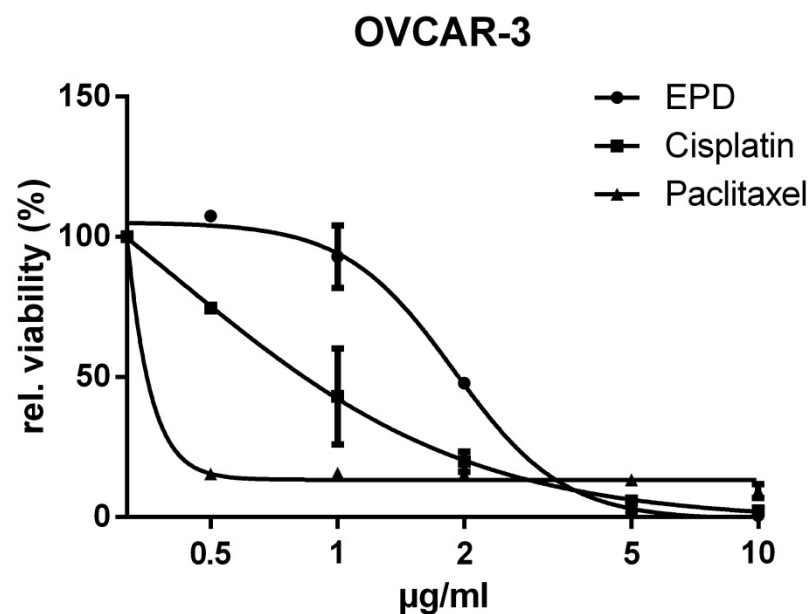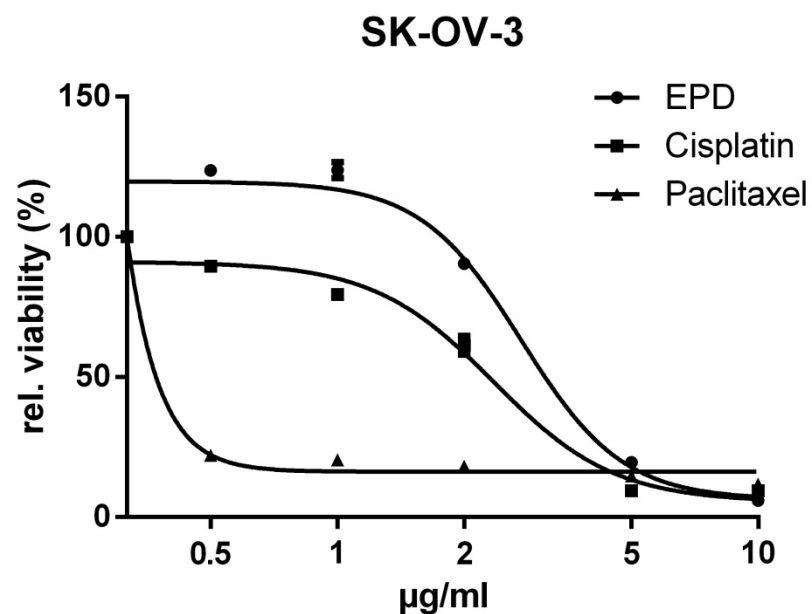

**Supplemental Figure 1**

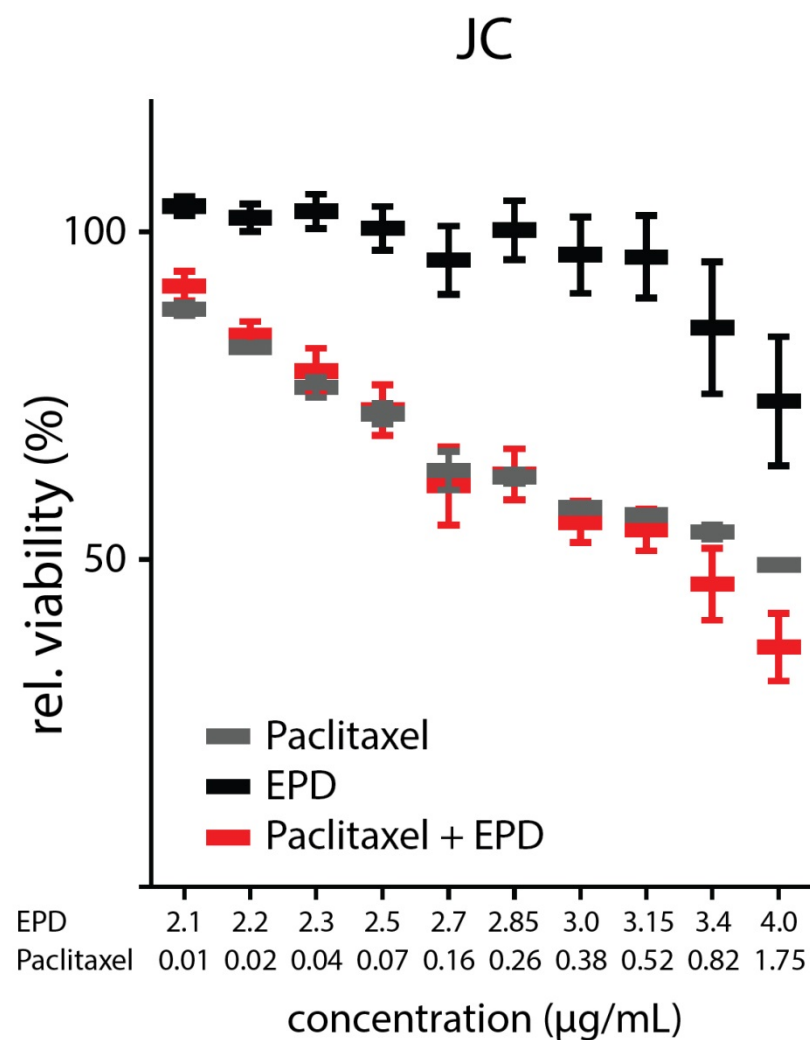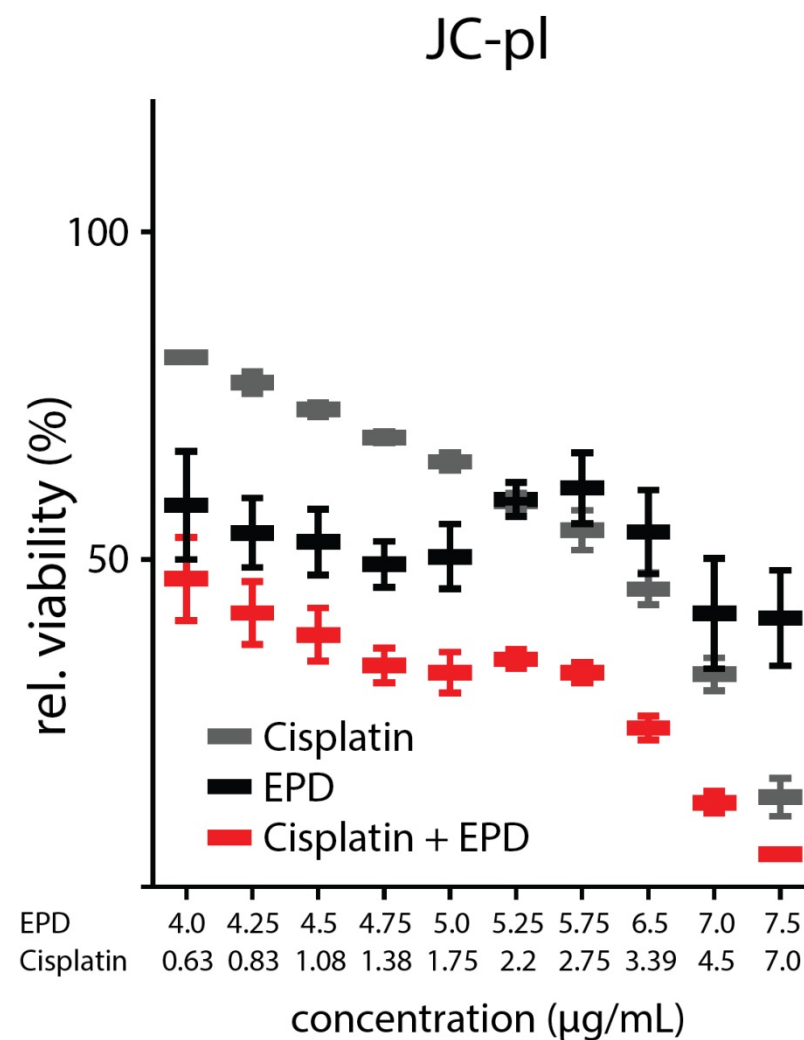

**Supplemental Figure 2**

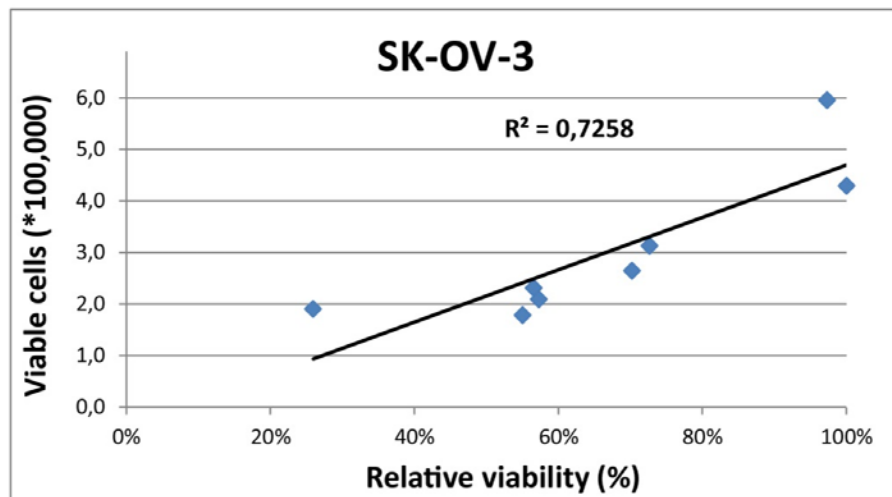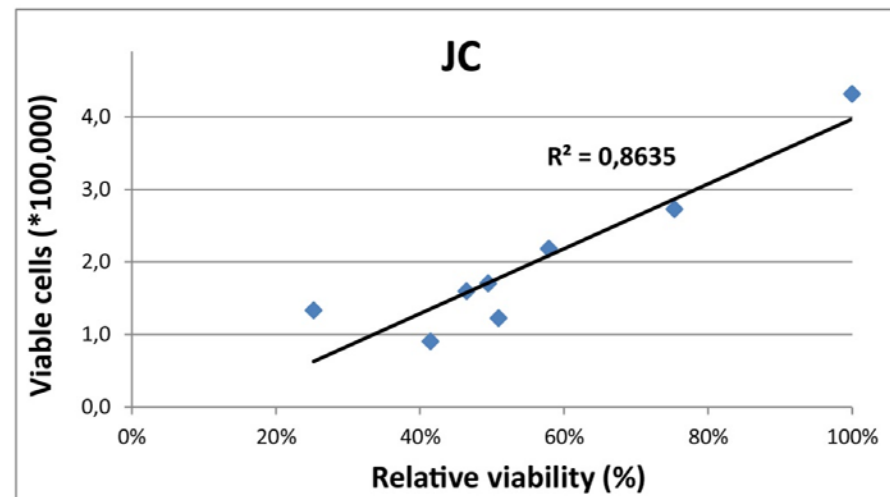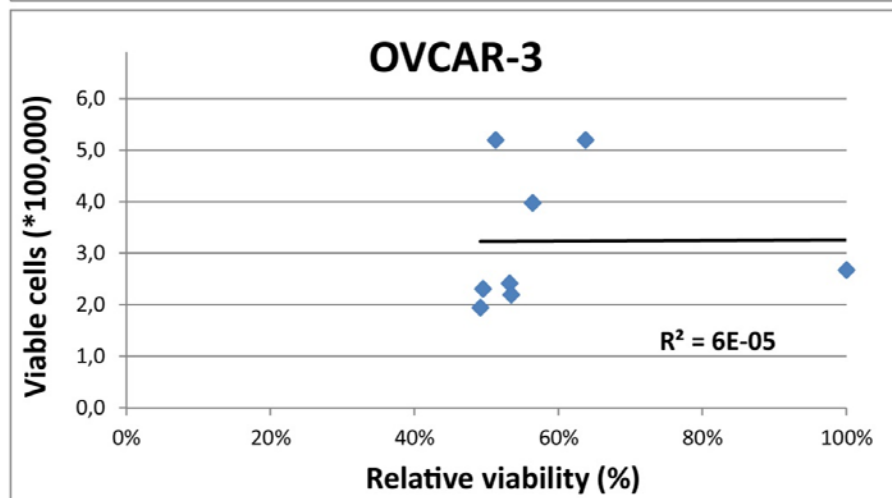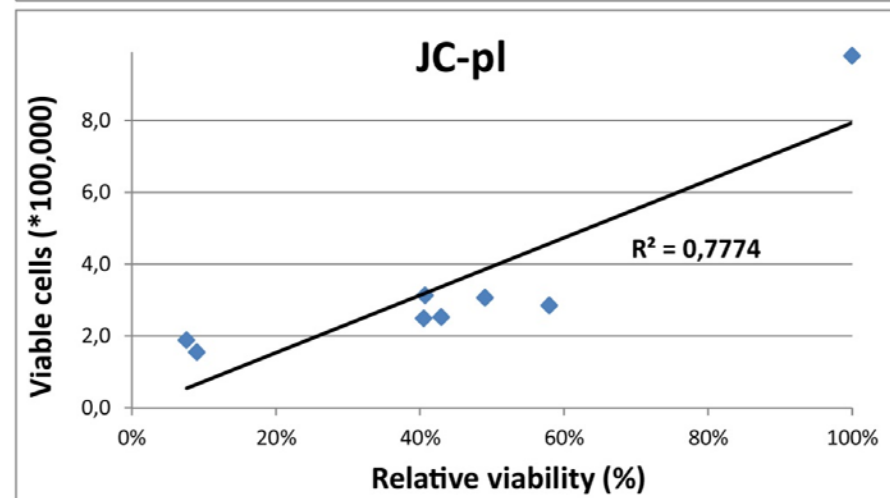

**Supplemental Figure 3**

Supplement: Additional file 1: — Figure S1. Dose response curves for EPD, cisplatin and paclitaxel for the four cell JC, JC-pl, OVCAR-3 and SK-OV-3. Figure S2. Observed relative viability for JC and JC-pl after treatment with single drug and combinations of EPD and paclitaxel (JC) and EPD and cisplatin (JC-pl). Figure S3. Percentage of viable cells per mL plotted against the relative viability for SK-OV-3, JC, OVCAR-3 and JC-pl. [file 13046_2015_157_MOESM1_ESM.pdf]
